# Update on Superficial Spindle Cell Mesenchymal Tumors in Children

**DOI:** 10.3390/dermatopathology8030035

**Published:** 2021-07-21

**Authors:** Philippe Drabent, Sylvie Fraitag

**Affiliations:** 1 Department of Pathology, Necker-Enfants Malades Hospital, 75015 Paris, France; philippe.drabent@aphp.fr; 2 Faculty of Medicine, Sorbonne University, 75006 Paris, France; 3 Faculty of Medicine, University of Paris, 75006 Paris, France

**Keywords:** mesenchymal tumors, children, skin, subcutis, connective tissue nevus, plaque-like CD34-positive dermal fibroma, fibrous hamartoma of infancy, lipofibromatosis, lipofibromatosis-like neural tumor, plexiform myofibroblastoma, *NTRK*, *RET*, *RAF*1, *BRAF*

## Abstract

The diagnosis of cutaneous and subcutaneous spindle cell neoplasms in children is often challenging and has potential therapeutic and prognostic implications. Although correctly diagnosing dermatofibrosarcoma protuberans and infantile fibrosarcoma is paramount, pathologists should not ignore a number of diagnostic pitfalls linked to mostly rare tumors with completely different clinical outcomes. In the last decade, a spectrum of novel entities has been described; information from molecular biology has helped to shape this new landscape for spindle cell tumors. Here, we review the most noteworthy neoplasms in this spectrum, with a focus on their histological similarities: fibroblastic connective tissue nevus, medallion-like dermal dendrocyte hamartoma, or plaque-like CD34-positive dermal fibroma, which share features with fibrous hamartoma of infancy; lipofibromatosis and lipofibromatosis-like neural tumor; and plexiform myofibroblastoma, a recently described neoplasm that should be distinguished from plexiform fibrohistiocytic tumor. These tumors also have genetic similarities, particularly gene rearrangements involving *NTRK*3 or *NTRK*1. These genetic features are not only essential for the differential diagnosis of infantile fibrosarcoma but are also of diagnostic value for lipofibromatosis-like neural tumors. The more recently described *RET, RAF*1, and *BRAF* gene fusions are also discussed.

## 1. Introduction

Pediatric spindle cell mesenchymal neoplasms form a very diverse group of tumors with differing prognoses. Recently, a few entities in the skin and subcutis have been described in morphological and molecular terms. There has been particular interest in CD34-positive spindle cell neoplasms, in view of their importance as a differential diagnosis for dermatofibrosarcoma protuberans (DFSP). One of the first of these tumors to have been described was medallion-like dermal dendrocyte hamartoma (MLDDH) [1], a tumor with a peculiar clinical aspect characterized by a well-circumscribed erythematous atrophic plaque and (under the microscope) a dermal proliferation of CD34-positive, S100-negative spindle cells. This tumor was first considered to be a hamartoma but has since been classified as a neoplasm of fibroblastic lineage and not of dendrocytic lineage [2]. The term “plaque-like CD34-positive dermal fibroma” (PDF) has therefore been preferred in the more recent literature, and there is a tendency to consider MLDDH and PDF as one and the same entity. MLDDH/PDF shares clinical and pathological features with fibroblastic connective tissue nevus (FCTN), a variant of connective tissue nevus first characterized in 2012 by Fletcher and de Feraudy in a series of 25 cases [3]. Although lipofibromatosis (LPF) mainly involves the subcutis, it also contains a spindle cell component admixed with adipose tissue and can be a differential diagnosis for superficial spindle cell tumors in children. In 2016, a CD34-positive spindle cell neoplasm with an interesting, recurrent genetic anomaly was described: LPF-like neural tumor (LPF-NT), which was initially associated with *NTRK*1 gene fusions [4]. As indicated by its name, this tumor is very similar to LPF. It is usually located in the subcutis but infiltrates the surrounding adipose tissue, and it is composed of spindle cells arranged in fascicles. Immunohistochemically, LPF-NTs are positive for both CD34 and S100 protein. Importantly, LPF-NTs are characterized by gene fusions in the *NTRK* family of genes, or (less frequently) in the *RAF* family of genes [5] or *RET* [6]. The clinical course is benign in most but not all cases. The newest member of these pediatric spindle cell neoplasms is plexiform myofibroblastoma (PM), as described in 2020 by Papke and Fletcher [7]. This entity broadens the spectrum of superficial fibroblastic/myofibroblastic tumors and is particularly similar to plexiform fibrohistiocytic tumor (PFHT). However, the two must be distinguished because PFHT is a low-grade sarcoma and PM is benign.

Here, we review these interesting new entities and focus on their similarities and differences. We hope to demonstrate that most of these entities are part of clinical-pathological spectra. Despite valuable contributions from molecular pathology and genetics, a few gray zones nevertheless remain.

## 2. The “Connective Tissue Nevus/Medallion-Like Dermal Dendrocyte Hamartoma/Plaque-Like Cd34-Positive Dermal Fibroma/Superficial Fibrous Hamartoma of Infancy” Spectrum

**Figure 1:**
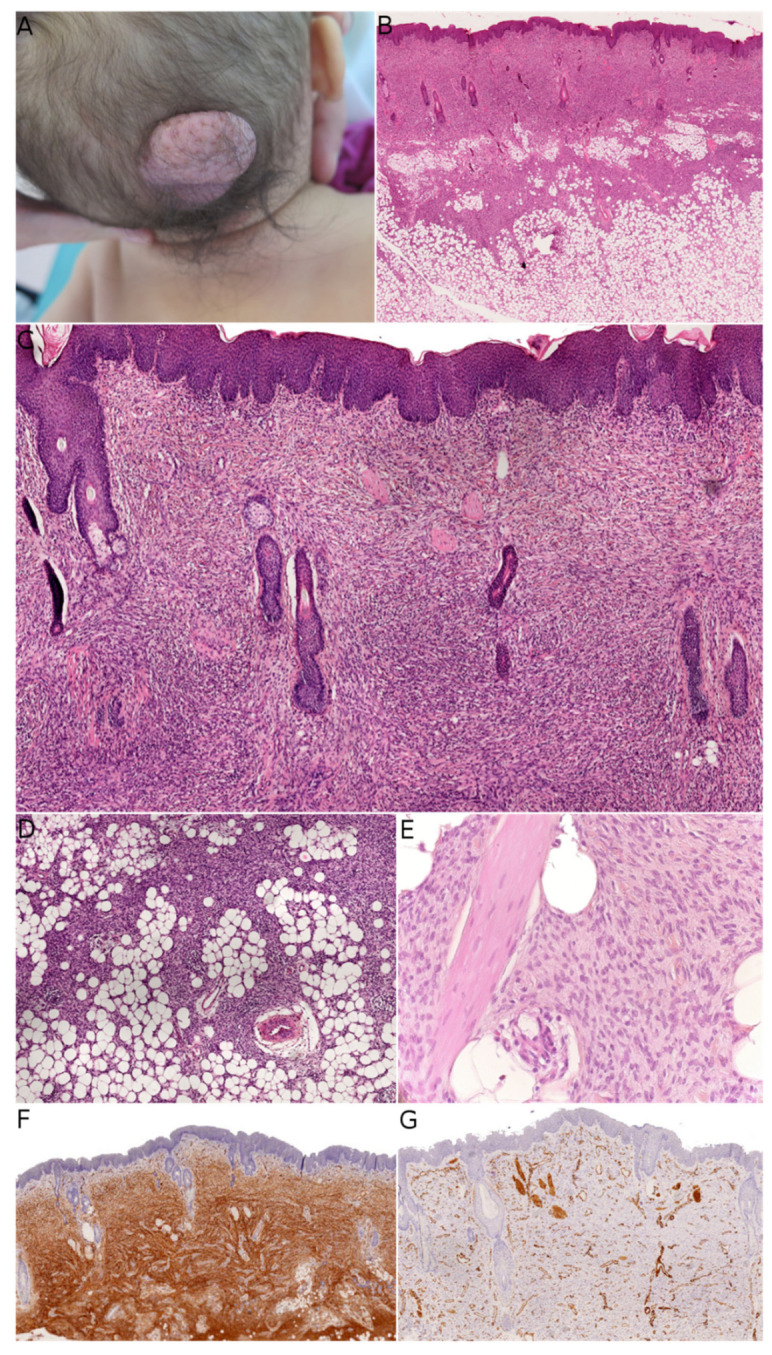
Fibroblastic connective tissue nevus. (**A**) Clinical presentation on the scalp of an infant (courtesy of the Department of Maxillofacial Surgery, Necker-Enfants Malades hospital, Paris, France). (**B**) Low-power view of the lesion showing infiltration of the dermis and subcutis (HE ×50). (**C**) Short intersecting fascicles surrounding the appendages and epidermal hyperplasia (HE ×100). (**D**) Extension into the subcutis (HE ×100). (**E**) High-power view showing the bland morphology of the spindle cells (HE ×200). (**F**) Diffuse positivity for CD34 (×50). (**G**) In this case, smooth muscle actin was negative, with internal controls on vessels and smooth muscles (×50).

Fibroblastic connective tissue nevus (FCTN) was described in 2012 in a series of 25 cases. This variant of connective tissue nevus has some clinical and histological particularities [3,8]. It typically appears during the first decade of life as a slowly growing, painless, plaque-like, or nodular skin lesion, and is mainly located on the trunk, head, and neck and less frequently on the limbs. Girls are more often affected than boys (sex ratio: 0.5–0.6). The lesions range from 0.18 to 2.0 cm in size, and FCTN is rarely diagnosed on the basis of clinical signs alone. Histologically, FCTN is a poorly circumscribed dermal lesion that arises in the reticular dermis and extends to the superficial subcutis. In 70% of cases, there is overlying papillomatosis of the epidermis. In about 60% of cases, abnormally superficial adipocytes are seen in the reticular dermis. The tumor is composed of short, intersecting fascicles of bland, spindle-shaped fibroblasts/myofibroblasts with weakly eosinophilic cytoplasm and elongated nuclei with no atypia and no mitoses. It extends between the collagen bundles, around the appendages and into the subcutaneous septa. The most useful immunostaining marker is CD34, which is positive in 87% of cases—albeit often weakly and in focal sites. Smooth muscle actin (SMA) is weakly and focally present in less than 50% of cases, and S100 is always absent (Figure 1).

The main differential diagnoses for FCTN are (i) plaque-stage DFSP and (ii) the fibroblast-predominant type of PFHT. DFSP differs from FCTN by its storiform architecture; the absence of epidermal papillomatous hyperplasia; the presence of a grenz-zone beneath the epidermis; the atypical nuclei; and the strong, widespread CD34 staining. If tested for, the presence of a *COL*1*A1–PDGFB* fusion transcript is pathognomic for DFSP. Furthermore, it should be borne in mind that other gene rearrangements have been found in the few cases of DFSP lacking the t(17; 22) translocation [9]. PFHT is discussed in more detail below, and its clinical features (an acral site) and morphological features (osteoclast-like giant cells or other histiocytoid component) will be of assistance in the differential diagnosis.

FCTN broadens the spectrum of connective tissue nevi (CTN), which are otherwise classified with regard to their most abundant component: collagen, elastin, or proteoglycans. In each of these categories, various entities have been described and differ in their clinical characteristics: inherited vs. acquired lesions, and an association with a genetic disorder (Buschke–Ollendorff syndrome, proteus syndrome, tuberous sclerosis complex, or multiple endocrine neoplasia type 1) [10]. A mixed pattern (in which both collagen and elastin are more abundant) appears to be more frequent than initially thought [8]. In line with this observation by Saussine et al., we suggest that the “connective tissue nevi spectrum” might be even broader and overlap with other entities.

**Figure 2:**
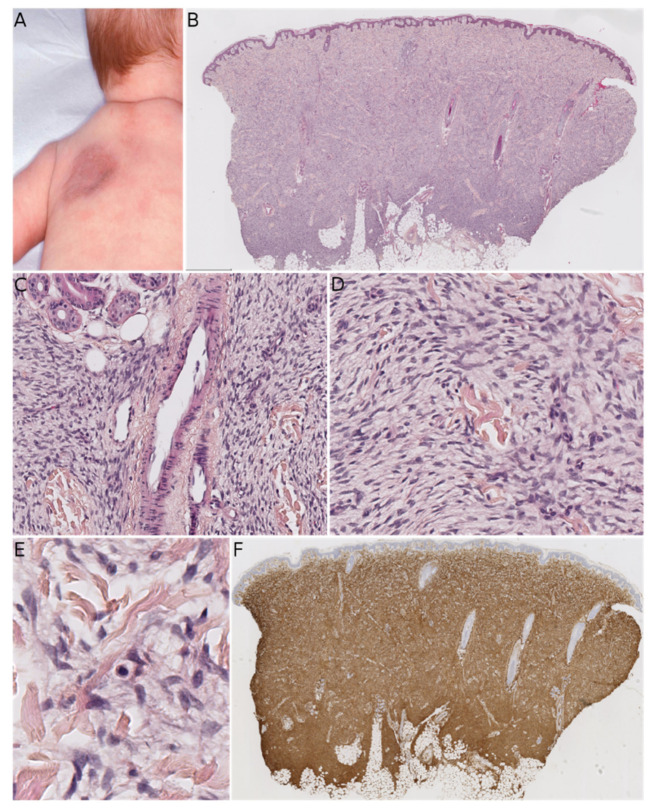
Medallion-like dermal dendrocyte hamartoma (MLDDH)/plaque-like CD34-positive dermal fibroma (PDF). (**A**) Clinical presentation as a medallion-like lesion on the upper back of an infant (courtesy of the Department of Maxillofacial Surgery, Necker-Enfants Malades hospital, Paris, France). (**B**) Low-power view of the lesion showing dermal location and slight extension into the subcutis (HES ×50). (**C**) Venules with dilated lumens (HES ×100). (**D**) Presence of both spindle cells and more ovoid cells (HES ×200). (**E**) High-power view showing the bland morphology of the spindle cells and a mast cell in the center of the picture (HES ×400). (**F**) Diffuse positivity for CD34 (×50).

MLDDH was the first of these entities to be described, in a series of three cases in 2004 [1]. The clinical presentation was characteristic: a medallion-like, well-circumscribed, brownish, erythematous lesion with a finely wrinkled, atrophodermic surface. The lesions were initially described as congenital, situated on the neck and the upper part of the trunk, and affecting girls. Some subsequently reported cases of MLDDH occurred in boys, appeared shortly after birth, or affected the limbs [11,12,13,14]. In 2010, Kutzner and colleagues described similar lesions on the limbs in four adults and a lesion of the neck in a 9-year-old boy [2]. The name “plaque-like CD34-positive dermal fibroma” (PDF) was suggested for these lesions because they appeared after birth and were more like tumors than hamartomas. The investigators also argued that dendrocytes are known to proliferate during wound healing and that PDF/MLDDH might be reactive lesions related to trauma. Histologically, MLDDH and PDF are indistinguishable [15]. The lesion consists of a superficial dermal spindle and sometimes ovoid cell proliferation, mainly occupying the reticular dermis but occasionally infiltrating the subcutis. The degree of epidermal atrophy is variable, and elastic fibers are often diminished or fragmented in the lesion. The stroma may contain mast cells and/or venules with a dilated lumen. The proliferating cells stain positive for CD34 (Figure 2). In their series of PDF cases, Kutzner et al. described a specific but inconstant pattern, with the fibroblasts in the upper part of the lesion oriented vertically to the epidermis and those in the lower part of the lesion oriented horizontally. The investigators also noted that PDF in adults never extended into the deep dermis and subcutaneous septa. Given this slight morphological distinction and the difference in clinical presentation between adults and children, it is still not clear whether PDF and MLDDH are one and the same lesion or if MLDDH is a congenital/infantile variant of PDF. It is noteworthy that MLDDH tends to extend into the subcutis, making it very challenging to differentiate between MLDDH and FCTN, as recently emphasized in a few case reports [15,16]. Both lesions may show slight infiltration clinically, although FCTN may be more irregular in shape [16]. Other differential diagnoses of MLDDH/PDF include dermatomyofibroma (which rather presents in young adults, shows fascicles preferentially oriented parallel to the epidermis, and is SMA-positive), non-pigmented cellular blue nevus (which is easily ruled out by immunophenotyping), plaque-like superficial neurofibroma (again, easily ruled out by immunophenotyping), and CD34-positive sclerotic fibroma of the skin (also known as “plywood fibroma”, featuring a more nodular or stellate-like silhouette with characteristic clefts; it is extremely rare in children and is mainly associated with Cowden syndrome).

**Figure 3:**
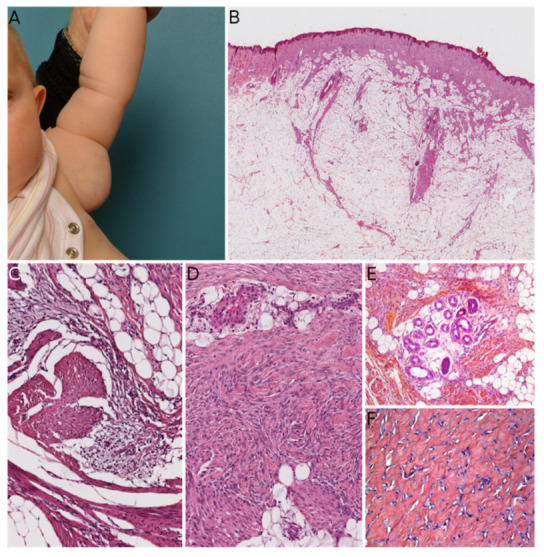
Fibrous hamartoma of infancy (FHI). (**A**) Typical clinical presentation as an axillary mass (courtesy of the Department of Maxillofacial Surgery, Necker-Enfants Malades hospital, Paris, France). (**B**) Low-power view of the lesion showing location in the subcutis and extension of the spindle-cell component into the dermis (HE ×50). (**C**) Presence of the three typical components (mature fibrous tissue, mature adipose tissue, immature mesenchymal tissue) (HE ×100). (**D**) In some cases, the immature mesenchymal tissue is not visible (HE ×100). (**E**) Peri-eccrine extension of the lesion (HES ×100). (**F**) Hyalinized zone reminiscent of giant cell fibroblastoma (HES ×100).

FCTN, MLDDH, and PDF can all contain cells that appear histologically similar to the fibroblastic component of fibrous hamartoma of infancy (FHI). Both the clinical and histological features must be examined when differentiating between FHI and the above-mentioned lesions. FHI arises in infants and young children—typically within the first two years of life—and is usually located in the axilla, upper limbs, and upper back [17,18]. It presents as a subcutaneous mass rather than a plaque, which is an important difference vs. MLDDH/PDF and some FCTNs. Histologically, it is essential to look for the three characteristic components of FHI: mature fibrous tissue, mature adipose tissue, and immature mesenchymal tissue (Figure 3). These three components are almost always present; if all components are seen, no other diagnosis should be sought. However, one or two of these components may be missing in biopsies or small samples. When the fibrous component alone is seen, misdiagnosis is possible. Furthermore, some FHI extend into the deep dermis and show a peri-eccrine and perivascular arrangement of bland spindle cells similar to those seen in MLDDH/PDF and FCTN [19]. An additional pseudoangiomatous pattern has been described in about half of cases and may be of diagnostic value [20]. Hyalinized zones with “cracking” artifacts mimicking giant cell fibroblastoma have also been observed, and the absence of a *PDGFB* gene rearrangement in these areas argues in favor of an FHI [18]. In the same report, the investigators discussed two interesting cases with sarcomatous-like foci, hyperdiploid or near tetraploid karyotypes, and segmental loss of heterozygosity (1p, 10p, 11p, chromosome 14, 22q). Amputation was decided in one of these cases, in view of extensive local disease and sarcomatous histological features. The investigators concluded that FHI is a complex neoplasm rather than a hamartoma. This is further supported by the results of Park et al. who found consistent *EGFR* exon 20 insertion/duplication mutations in FHI [21]. In difficult-to-diagnose cases, sequencing of *EGFR* exon 20 might be of value for diagnosing or ruling out FHI. Moreover, this raises the possibility of using tyrosine kinase inhibitors to treat large, non-resecable or relapsing FHI.

All the above-discussed lesions have similarities: they can be seen during infancy and can affect the upper back/trunk or upper limbs. Some present mostly as plaque-like lesions (MLDDH/PDF), some present as masses (FHI), and some present as both (FCTN). Under the microscope, all these lesions may show a proliferation of fibroblastic/myofibroblastic cells arranged in fascicles in the reticular dermis. For these reasons, it is important to consider all the available data (i.e., clinical; morphological; and, if necessary, molecular) when confronted with a lesion that belongs to this spectrum. In most cases, the presence of one or more of the characteristics described above will help the pathologist to make the right diagnosis (see Table 1). The most troublesome differential diagnosis is plaque-like DFSP, and therefore all equivocal cases should be screened for the *COL*1*A*1*-PDGFB* gene rearrangement. The literature data show that the combination of RT-PCR and FISH is the most sensitive diagnostic method [22]. For cases in which the *COL*1*A*1*-PDGFB* rearrangement is not found, our experience shows that it is best to take a closer look at the morphology: if there is any suspicion of DFSP, other gene rearrangements should be sought using RNA sequencing (RNA-seq) or next-generation sequencing techniques; if the morphology is not suggestive of DFSP, the most likely diagnosis will fall within the “FCTN-MLDDH/PDF” spectrum. Unfortunately, a characteristic, recurrent genetic abnormality has not yet been evidenced in cases of acquired CTN or MLDDH/PDF.

## 3. The “Lipofibromatosis/Lipofibromatosis-Like Neural Tumor” Spectrum—The “Lipofibromatosis-Like Pattern”

**Figure 4:**
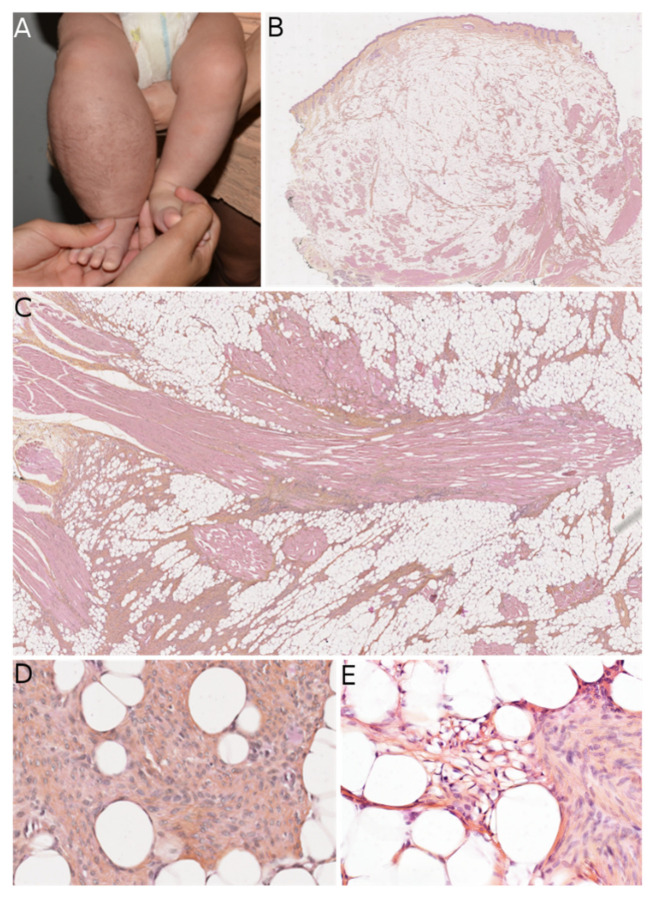
Lipofibromatosis (LPF). (**A**) Clinical presentation as a large mass of the right lower leg in an infant (courtesy of the Department of Maxillofacial Surgery, Necker-Enfants Malades hospital, Paris, France). (**B**) Low-power view of the lesion showing location in the subcutis (HES ×50). (**C**) Long fascicles of spindle cells admixed with adipose tissue (HES ×100). (**D**) High-power view showing the bland morphology of the cells (HES ×200). (**E**) Focus on cells with a single vacuole at the interface between the fibroblastic and the adipose components (HES ×200).

LPF was first described and named as such in 2000 [23], but this entity was already known and had been referred to by some as “infantile fibromatosis”, which can cause confusion. LPF arises mainly in infants but can be present at birth. The initial series reported by Fetsch et al. showed male predominance (sex ratio: 2.7). The main sites involved are the distal parts of the upper limbs, followed by the lower limbs and (more rarely) the trunk or head. Microscopically, LPF is an infiltrative, poorly-circumscribed lesion, consisting of mature adipose tissue and spindled fibroblastic cells with focal, fascicular growth that extends mainly into the septa of fat and skeletal muscle. The fibroblastic cells show limited cytologic atypia and low mitotic activity. Cells with a single vacuole can sometimes be seen at the interface between the fibroblastic component and the mature adipocytes, which is suggestive of a transition between the two components (Figure 4). Immunohistochemistry is of limited value; it can show foci positive for CD34, SMA, and (more rarely) EMA. It is likely that the rare initial reports of a positivity for S100 protein correspond to another tumor (discussed below) that was unknown at that time. Recurrences are frequent.

LPF can be hard to differentiate histologically from FHI, especially in cases of FHI that lack a visible immature, mesenchymal component. Although immunohistochemistry is not helpful, LPF and FHI tend to occur in different sites.

**Figure 5:**
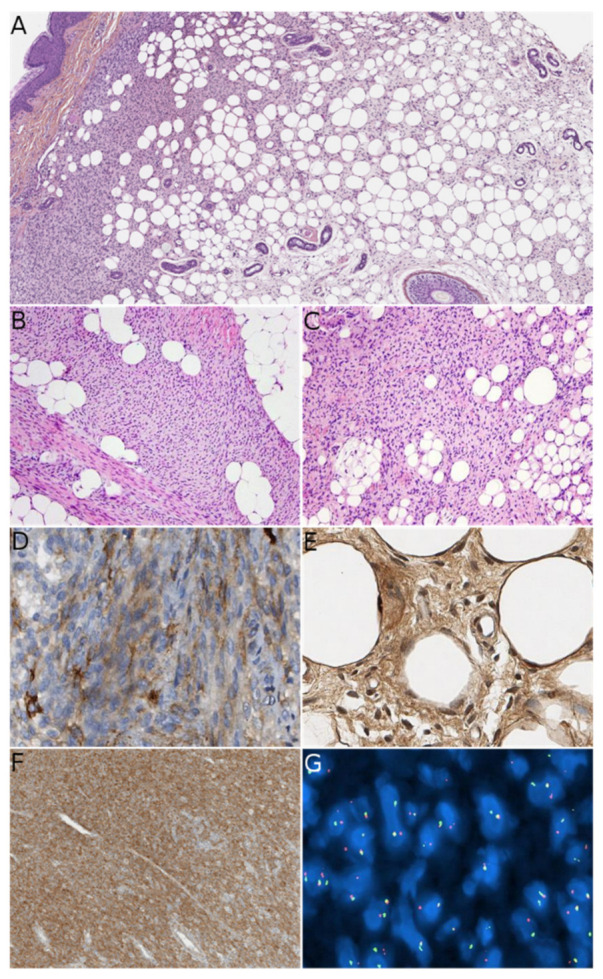
Lipofibromatosis-like neural tumor (LPF-NT). (**A**) Low-power view showing extension of the tumor in both the dermis and subcutis (HE ×50). (**B**) Spindle cells in fascicles admixed with adipose tissue (HE ×100). (**C**) Some areas can lack the typical fascicular growth (HE ×100). (**D**) Heterogeneous positivity for CD34 (×400). (**E**) Positivity for S100 (×400). (**F**) Diffuse positivity for NTRK1 (×200). (**G**) FISH showing fusion transcripts between *NTRK*1 and *LMNA*. (Courtesy of Marie Karanian, Department of Pathology, Centre Léon Bérard, Lyon, France).

Recently, a spindle cell tumor with some similarities to LPF has been identified in adults and children and named “lipofibromatosis-like neural tumor” (LPF-NT) [4]. Interestingly, most of these tumors were first diagnosed as low-grade malignant peripheral nerve sheath tumors (LG-MPNSTs) in adults, which emphasizes the morphological similarity between these entities. However, LG-MPNSTs are almost always seen in adult or adolescent patients with neurofibromatosis type 1 (NF1), whereas this is never the case for LPF-NTs. In our experience, LPF-NTs in children were mostly misdiagnosed as LPF. LPF-NT can affect infants, older children, adolescents, and young adults, with a possible predominance in infants and adolescents. There is no obvious sex predominance. The presentation of LPF-NT appears to vary with age, due to differences in fat distribution. The limbs are affected more often than the trunk, and there appear to be more cases involving the trunk in infants and the chest in pubescent girls. LPF-NT presents as a subcutaneous tumor with a greatest dimension of 1 to 6 cm. Microscopically, the tumor has an infiltrative growth pattern within subcutaneous fat and can extend into skeletal muscle in some cases. It is composed of dense spindle cells arranged in streaming fascicles. The tumor cells have weakly eosinophilic cytoplasm with indistinct borders, elongated nuclei with mild atypia and hyperchromasia, and inconspicuous nucleoli. In rare cases, scattered pleomorphic cells may be present. The mitotic activity is low, with less than two mitoses per 10 high-power fields (HPFs) in most cases, and rarely more than 8 mitoses per 10 HPFs. One of the most striking characteristics of these tumors is the dual positivity for S100 protein and CD34 (sometimes focal) (Figure 5). A small proportion of cases can be positive for SMA. Melanocyte markers (SOX10, HMB45, melan A), desmin, STAT6, and GFAP are absent. H3K27me3 expression is retained, which can be useful in the differential diagnosis vs. MPNST. Local recurrences are possible, but metastases have not been reported. The key diagnostic element is the presence of recurrent gene fusions involving the *NTRK*1 gene, which encodes the TRKA receptor tyrosine kinase (RTK), the high affinity receptor for the nerve growth factor. The various fusion partners include LMNA, TPM3, and TPR [4]. None of these gene rearrangements have been found in LPF [24]. However, no *NTRK*1 gene partner can be identified in a subset of LPF-NTs [4,5]. Importantly, an anti-*NTRK*1 antibody (or a panTRK antibody) can be used to immunohistochemically screen for NTRK1 expression. If this test is negative, molecular techniques must be used. Since the first description of LPF-NT, other gene rearrangements have been identified in children; they involve *NTRK*3 (with *EML*4 or *KHDRBS*1 as partners) [5,25,26], *RAF*1, *BRAF* [5], or *RET* (with *CCDC*6 or *NCOA*4 as partners) [6]. These interesting molecular results require further explanation.

In a study reported in 2018, Suurmeijer et al. described a new group of spindle cell tumors with S100 and CD34 co-expression and recurrent gene fusions. In their series of 25 cases, 8 were located in subcutaneous tissue in children. Interestingly, there were four other pediatric cases involving visceral organs (the stomach and the rectum) or bones (the maxilla and the mandible). The remaining cases were in adults and featured a variety of superficial and deep sites. All the tumors had similar features: monomorphic cytology; stromal and perivascular hyalinization; immunopositivity for S100 and CD34; and *RAF*1, *BRAF*, or *NTRK*1/2 fusions. Hence, it is likely that LPF-NT belongs to a wider group of spindle cell tumors sharing these characteristics. Moreover, some tumors showed possible signs of malignancy: scattered pleomorphic and/or multinucleate cells, highly cellular fascicular growth, or a primitive appearance. In fact, an 18-year-old patient in Suurmeijer et al.’s series died of metastatic disease, and other patients had metastases (in the lung and other sites) or disease recurrence. This aggressive clinical course was seen both for superficial and deep tumors, and in patients as young as 4 years old. It is most likely that tumors defined by *RAF*1, *BRAF*, or *NTRK*1/2 fusions form a spectrum whose behavior can vary from benign to malignant.

In 2019, Antonescu et al. reported on six cases with tumors that resembled LPF-NT but harbored *RET* gene rearrangements. Five of their cases occurred in children (including four infants): three of these were diagnosed as LPF-NT, one was diagnosed as an “infantile fibrosarcoma-like” tumor (involving the chest wall), and the last was diagnosed as infantile fibrosarcoma/cellular mesoblastic nephroma (involving the kidney with bilateral lung and brain metastases). Of the three cases of LPF-NT, one was a superficial tumor of the ankle, one was a deep tumor of the foot, and the last arose in the abdominal wall. None of the LPF-NTs recurred, although the tumor had an aggressive clinical course in the two cases with malignant histological features. These interesting results broadened the spectrum of spindle cell tumors with recurrent translocations and emphasized two important features of this group: (i) the predominant value of molecular characteristics (i.e., recurrent gene fusions) in the classification of these tumors with a wide range of morphologies (from LPF-NT to infantile fibrosarcoma to MPNST-like tumors), and (ii) the probable continuum in the degree of malignancy.

This second concept was already foreseen in the first description of LPF-NT by Agaram et al., who described a case of superficial LPF-NT of the leg in a young woman who developed lung metastases [4]. This tumor had the *LMNA–NTRK*1 fusion, and its possible identity as a malignant counterpart of LPF-NT was mentioned by the researchers at the time.

The varying degrees of aggressiveness within this group of tumors warrant further investigation. To the best of our knowledge, no ancillary techniques (whether immunohistochemical or molecular) appear to be of value in this respect. At present, only the morphology seems to be related to the tumor’s aggressiveness: elevated cellularity, presence of pleomorphic and/or multinucleate cells, and elevated mitotic activity are signs that should alert the pathologist and the clinician.

Besides the very similar FHI and LPF, the differential diagnoses of LPF-NT also include other entities, the most important of which is calcifying aponeurotic fibroma (CAF). This lesion is a superficial, slow-growing, ill-circumscribed tumor of the hands or feet that arises in children and adolescents. Histologically, CAF has a typically two-phase morphology that combines moderately cellular areas (with fibroblastic cells arranged in fascicles) and partly calcified nodules (with a fibrocartilage appearance, small epithelioid fibroblasts, and osteoclast-like giant cells). In 2016, a recurrent *FN*1*–EGF* fusion was found in eight out of nine cases, either by RNA-seq, RT-PCR, or FISH [27]. This fusion results in overexpression of epidermal growth factor (EGF), which is detected using immunohistochemistry in all cases, even when a gene fusion cannot be detected. Interestingly, in a report in 2019, the *FN*1*–EGF* fusion was found in four cases of apparently typical LPF that recurred as CAF [24]. In this study of 20 LPF cases, the other fusions involved genes that encode ligands for the EGF receptor (*EGF*, *TGFA*, *HBEGF*) or that encode RTKs as 3′-partners (*ROS*1, *PDGFRB*, *RET*). The researchers mentioned that these findings were strongly suggestive of a link between LPF and CAF; in some cases, LPF might correspond to “early” CAF characterized by a prominent adipocytic component and no calcification. It remains to be established whether the cases with a *FN*1*–EGF* fusion or another fusion constitute the same entity or whether the cases with *FN*1*–EGF* fusions are CAF and the cases with other fusions are LPF. However, this interesting study highlighted potential genetic variability in LPF, with a diverse range of gene fusions and proximity to CAF. Lastly, the differential diagnoses for LPF and LPF-NT in children include DFSP, which can be distinguished by its negativity for S100 protein and the presence of the characteristic gene fusions.

The most important features to look for when confronted with an “LPF-like lesion” are summarized in Table 2.

## 4. Plexiform Myofibroblastoma

**Figure 6:**
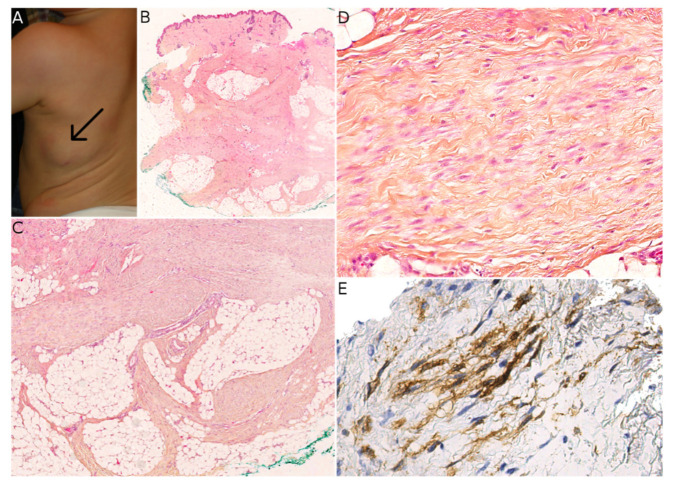
Plexiform myofibroblastoma (PM). (**A**) Clinical presentation as a sub-scapular subcutaneous mass in an infant (courtesy of the Department of Maxillofacial Surgery, Necker-Enfants Malades hospital, Paris, France). (**B**) Low-power view of the lesion showing location in the subcutis (HES ×50). (**C**) Typical plexiform architecture with extension into the subcutaneous septa (HES ×100). (**D**) High-power view showing the bland morphology of the cells and the characteristic collagenous stroma (HES ×200). (**E**) Positivity for smooth muscle actin in the spindle cells (SMA ×200).

Plexiform myofibroblastoma (PM) is a newly described entity. In a series of 36 cases, Papke et al. characterized this tumor; it had a broad age range and no male or female predominance (19 females and 17 males) [7]. PM can be congenital but may appear as late as the age of 50, although the median age at onset is 9.5 years. A total of 24 of Papke et al.’s 36 patients were children. In the pediatric population, the tumor was located (i) mostly in the neck and upper back; (ii) to a lesser extent in the lower back, axilla, chest wall and abdominal wall; and (iii) in one case in the left lower leg. The lesions were multifocal in three cases (all below the age of 2) and extended to the occiput in one case. Interestingly, two of the multifocal cases were brothers, raising the question of a genetically inherited anomaly (though not discussed by Papke et al.). None of the pediatric cases—even those with positive margins—showed any recurrence of the disease after surgical resection. Grossly, PM has a tan/white cut surface and ranges from 0.6 to 4 cm in size (in pediatric cases). Histologically, it is composed of fascicles in a plexiform pattern, which extend within the reticular dermis and subcutis. The tumor cells are fibroblastic/myofibroblastic, with a weakly eosinophilic, elongated cytoplasm and ovoid to tapered nuclei with no atypia. The characteristic collagenous stroma is reminiscent of mammary-like myofibroblastoma, with at least focal hyalinization in 35% of cases. Some cases show nodular fasciitis-like areas. The stroma may be focally myxoid. Mitoses are rare, with no more than 4 per 2 mm^2^. There is no necrosis. The most frequently positive immunohistochemical markers are SMA (in 84% of cases), CD34 (in 54%), and desmin (in 43%) (Figure 6). The tumor is also negative for S100 protein, and there is no nuclear translocation of beta-catenin. To date, no recurrent genetic alterations have been identified. Only one case had a missense mutation in *FGFR*2, and another had a probable germline mutation in both *MUTYH* and *BRIP*1; the significance of these genetic abnormalities remains unclear.

There are many potential differential diagnoses for PM. The closest mimic is the fibroblastic variant of plexiform fibrohistiocytic tumor (PFHT), a superficial tumor of children and young adults (median age at onset: ≈15) that occurs mainly in the upper limbs but also in the axilla, back, neck, and chest wall [28]. Like PM, PFHT has a characteristic plexiform architecture. Most cases have a fibrohistiocytic or mixed fibroblastic–fibrohistiocytic morphology. Cases with a predominant fibroblastic component are the hardest to differentiate from PM, especially if no osteoclast-like giant cells or histiocytoid cells are present. CD68 immunostaining is useful for highlighting the histiocytoid component. PM is usually SMA-positive (which is not helpful for the differential diagnosis), and CD34 is usually negative. As discussed by Papke et al., it is possible that cases of purely fibroblastic PFHT are in fact PM. Interestingly, there are no reports of recurrence or metastasis in purely fibroblastic PFHT [7]. It is important to differentiate between PM and PFHT; the former is benign and does not seem to recur in children, whereas the latter is a low-grade sarcoma with metastatic potential.

Other differential diagnoses for PM lack the typical plexiform pattern and can thus be ruled out quite easily: desmoid fibromatosis, nodular fasciitis, dermatomyofibroma, plaque-like myofibroblastic tumor, FCTN, and FHI.

## 5. Conclusions

Superficial spindle cell mesenchymal tumors form a diverse group of lesions with benign and malignant entities that are often very similar clinically and/or histologically. In children, the patient’s age; the lesion site; the presentation as a mass, nodule, or plaque; and the location in the dermis or subcutis are important features that will guide the dermatologist and the pathologist. The newest entities encompass two main histologic patterns.

The first pattern encompasses dermal, CD34-positive spindle cell lesions that can extend to the subcutis. The major differential diagnosis is DFSP. The distinction between FCTN, MLDDH/PDF, and the superficial variant of FHI can be challenging for the pathologist and the clinical utility of making this distinction is not obvious or well characterized. Indeed, all these entities probably fall within a common spectrum, which corresponds to a kind of fibroblastic-rich connective tissue nevus. Regardless of the type, all these lesions are benign and will be cured by excision with narrow margins. DFSP is the exception since it has an intermediate clinical behavior with frequent local recurrence. A wide excision is necessary, better achieved by either Mohs or slow-Mohs micrographic surgery [29]. A close follow-up is mandatory since rare cases of malignant transformation have been reported.

The second pattern encompasses subcutaneous lesions admixed with adipocytes and that are similar to LPF. It is important to consider other diagnoses when confronted with an LPF-like lesion: we have seen that CAF can mimic LPF and that this diagnosis can be confirmed by the presence of an *FN*1*–EGF* fusion. S100-protein-positive cases are likely to be LPF-NT and can be confirmed by the presence of gene fusions involving the *NTRK* family, the *RAF* family, or *RET*. In some relapsing cases or in overly superficial biopsies, a diagnosis of infantile fibrosarcoma could also be considered and then confirmed by the presence of the *ETV*6*–NTRK*3 gene fusion [30]. MPNSTs are extremely rare in children and should be diagnosed with great caution.

The latest tumor to have been identified is PM, which is characterized by its plexiform architecture. Given the benign clinical course of PM, it is essential to distinguish this entity from PFHT.

The use of molecular techniques must be encouraged whenever the morphological and clinical data are insufficient, and especially when one of the differential diagnoses is a malignant condition. This is true for DFSP, some non-resectable or relapsing cases of FHI, LPF-NT, and other tumors with gene fusions in *NTRK*, *RAF*, or *RET*. Although the presence of a gene fusion is not essential for malignancy, it is an important element of diagnostic information and may provide a rationale for treatment with RTK inhibitors (e.g., larotrectinib for *NTRK*-rearranged tumors, and anti-EGFR agents for FHI) [31,32].

## Figures and Tables

**Table 1:** The main clinical and pathological features of lesions from the “connective tissue nevus/medallion-like dermal dendrocyte hamartoma/plaque-like CD34-positive dermal fibroma/superficial fibrous hamartoma of infancy” spectrum and of dermatofibrosarcoma protuberans.

	Age	Sex	Site	Morphology	IHC	Genetic Abnormality
FCTN	<10 y	F > M	Trunk, head and neck, limbs	Poorly delimited, reticular dermis with extension into the subcutis; short bundles of (myo)fibroblasts	CD34+ weak, multifocal (87%) S100−	
MLDDH/PDF	Infants and older children	F > M	Neck, upper trunk, limbs	Poorly delimited, reticular dermis with possible extension into the subcutis; spindle and/or ovoid cells; variable epidermal atrophy, elastic fibers diminished	CD34+ S100−	
Superficial FHI	<2 y	M > F	Axilla, upper limbs, upper back	Subcutis, three components: mature fibrous tissue, mature adipose tissue, and immature mesenchymal tissue; possible pseudoangiomatous or hyalinized areas	Not helpful	*EGFR* exon 20 ins/dup
DFSP	Older children	M > F	Trunk, proximal areas of the limbs, head and neck	Diffuse infiltration of the dermis and subcutis; high cellularity; spindle cells in a storiform pattern	CD34+ S100−	*COL*1*A*1*-PDGFB*, other rare fusions

FCTN: fibroblastic connective tissue nevus; MLDDH: medallion-like dermal dendrocyte hamartoma; PDF: plaque-like CD34-positive dermal fibroma; FHI: fibrous hamartoma of infancy; DFSP: dermatofibrosarcoma protuberans.

**Table 2:** The main clinical and pathological features of “LPF-like” lesions.

	Age	Sex	Site	Morphology	IHC	Genetic Abnormality
FHI	<2 y	M > F	Axilla, upper limbs, upper back	Subcutis, three components: mature fibrous tissue, mature adipose tissue, and immature mesenchymal tissue; pseudoangiomatous or hyalinized areas may be found	Not helpful	*EGFR* exon 20 ins/dup
LPF	Infants	M > F	Upper limbs (more than lower limbs), trunk, head	Subcutis, poorly delimited, mature fat and spindled fibroblasts, single-vacuole cells at the interface	Not helpful	Possible multiple gene fusions involving RTK or RTK ligands
LPF-NT	All ages	M = F	Limbs > trunk	Subcutis, infiltrative, “MPNST-like”, possible scattered pleomorphic cells	CD34+ S100+ SOX10−	*NTRK*1/3, *RAF*1, *BRAF*, *RET* fusions
CAF	Children and adolescents	M > F (slight)	Hands, feet	Subcutis, aponeuroses, infiltrative growth, fibroblasts in fascicles, fibrocartilage-like nodules (sometimes absent), osteoclast-like giant cells (sometimes absent)	SMA+ (in most cases)	FN1–EGF

CAF: calcifying aponeurotic fibroma; FHI: fibrous hamartoma of infancy; LPF: lipofibromatosis; LPF-NT: lipofibromatosis-like neural tumor; RTK: receptor tyrosine kinase.
